# Role-Play versus Standardised Patient Simulation for Teaching Interprofessional Communication in Care of the Elderly for Nursing Students

**DOI:** 10.3390/healthcare10010046

**Published:** 2021-12-27

**Authors:** Alda Elena Cortés-Rodríguez, Pablo Roman, María Mar López-Rodríguez, Isabel María Fernández-Medina, Cayetano Fernández-Sola, José Manuel Hernández-Padilla

**Affiliations:** 1 Department of Nursing, Physiotherapy and Medicine, University of Almería, 04120 Almería, Spain; cra566@ual.es (A.E.C.-R.); mlr295@ual.es (M.M.L.-R.); isabel_medina@ual.es (I.M.F.-M.); cfernan@ual.es (C.F.-S.); j.hernandez-padilla@ual.es (J.M.H.-P.); 2 Health Sciences Research Centre, University of Almería, 04120 Almería, Spain; 3 Faculty of Health Sciences, Universidad Autónoma de Chile, Temuco 4810101, Chile; 4 Adult, Child and Midwifery Department, School of Health and Education, Middlesex University, The Burroughs, London NW4 4BT, UK

**Keywords:** interprofessional communication, nursing, role-play, simulation, standardised patient

## Abstract

This study aims to describe and compare the effects of standardised patient simulation and role-play in the acquisition and retention of interprofessional communication in elderly care competence amongst nursing students. In this controlled clustered randomised trial, 121 nursing students attended a workshop on interprofessional communication in elderly care using role-play or standardised patient simulation. The study was conducted between September 2017 and February 2018. Participants’ knowledge, self-efficacy and communication skills were assessed using a simulated scenario at pre-test, post-test and 6-week follow-up points. Between-subject and within-subject differences were measured using counts and proportions of participants who achieved competence. Regardless of the strategy applied, a significant improvement in knowledge, skills, self-efficacy and overall interprofessional communication competence was found between pre-test and post-test. Moreover, there were significant differences between pre-test and follow-up for all the studied variables, but no differences were found between post-test and follow-up. Lastly, when comparing the success rates of both strategies, no significant differences were observed (*p* > 0.05). In conclusion, standardised patient simulation and role-play have been shown to promote an improvement on knowledge, self-efficacy and interprofessional communication skills in nursing students, although it is not possible to state which strategy is the most adequate for teaching this competency.

## 1. Introduction

Interprofessional communication is understood to be a collaborative approach to the exchange of information between professionals in an open and responsive manner [1]. Older patients have complex health needs and they require coordinated interprofessional teams that give them holistic care planning [2]. Research suggests that effective interprofessional communication in elderly care can reduce pain, falls, depression, length of hospital admissions and mortality amongst older people [3]. Moreover, competent interprofessional communication has been associated with less stress in the workplace and higher satisfaction levels amongst older adults and healthcare professionals [4,5,6,7]. However, a large percentage of sentinel events occur due to interprofessional communication failures [8]. Ineffective interprofessional communication leads to delays in treatment, medication errors, patient injury and even death [9,10,11]. Furthermore, interprofessional miscommunication affects nurses’ interactions, integrity and well-being, which could in turn influence patients’ outcomes [11,12]. Consequently, interprofessional communication is considered to be pivotal to safe elderly care and it is included in most undergraduate nursing programmes internationally [13]. However, nursing students continue to report difficulties when communicating with other healthcare professionals [14,15]. In fact, they do not feel prepared to confront other healthcare professionals or to face controversial situations [16]. Furthermore, they report having problems with communication during clinical handovers [15] and their lack of assertiveness is a recurrent patient safety issue [17]. This situation makes them feel anxious and uncomfortable, affecting their clinical performance [18].

### Background

In line with the European Qualification Framework for lifelong learning and other classic taxonomies of learning and outcomes, being competent requires proving one’s ability to appropriately use knowledge, skills and attitudes [19,20,21]. In addition, Bandura [22] suggested that the acquisition of knowledge and skills does not lead to competence, unless individuals achieve a high level of self-efficacy, which can be defined as one’s belief in their ability to perform effectively.

In Spain, nursing curricula include general, transversal and specific competencies that must be attained by all qualified nurses. Interprofessional communication is defined as a transversal competency focused on ensuring that nursing students learn how to communicate with other colleagues and to work as part of a healthcare team that looks after older adults [23]. Globally, interprofessional communication is a core component in most nursing curricula and efforts have been made in order to design and implement teaching strategies that promote nursing students’ acquisition of competence [24,25,26,27]. Some of these strategies include experiential teaching and learning methods, which aim at fostering the acquisition of competence in terms of knowledge, skills and self-efficacy [28]. Amongst experiential teaching and learning methods, standardised patient simulation and role-play have proven to promote the acquisition of knowledge, skills and self-efficacy in interprofessional communication [29,30].

Standardised patient simulation has been recommended as a mean to increase patient safety and to reinforce interprofessional communication skills [31]. In addition, this method can bring a greater realism to the training as it involves actors performing as patients or healthcare professionals [29]. Standardised patient simulation requires small teaching groups, specific facilities and trained actors, which can prove difficult in low-budget contexts [32,33,34]. In contrast, role-play requires less organisation time and financial investment [32,33] as it involves students playing specific roles to work on solving a given situation [35]. Moreover, some nursing students do not enjoy role-play because they perceive the scenarios to be artificial and they feel ashamed while being observed by their peers [36].

Currently, few studies have compared the effects of standardised patient simulation or role-play on nurses’ and nursing students’ competence to communicate with other healthcare professionals [18,37,38,39]. Studies comparing role-play versus traditional strategies, such as lectures and class discussions [18,38,39], observed an improvement in students’ competence in terms of knowledge and skills. On the other hand, studies applying standardised patient simulation [20,37,40] showed inconsistent results in relation to students’ self-efficacy and skills. However, to the best of our knowledge, the effects of these strategies (standardised patient simulation vs role-play) on nursing students’ competence in interprofessional communication in elderly care have not been compared.

The aim of this study was to describe and compare the effects of two teaching strategies (standardised patient simulation vs role-play) in the acquisition and retention of interprofessional communication in elderly care competence amongst nursing students.

## 2. Materials and Methods

### 2.1. Design

**Figure 1:**
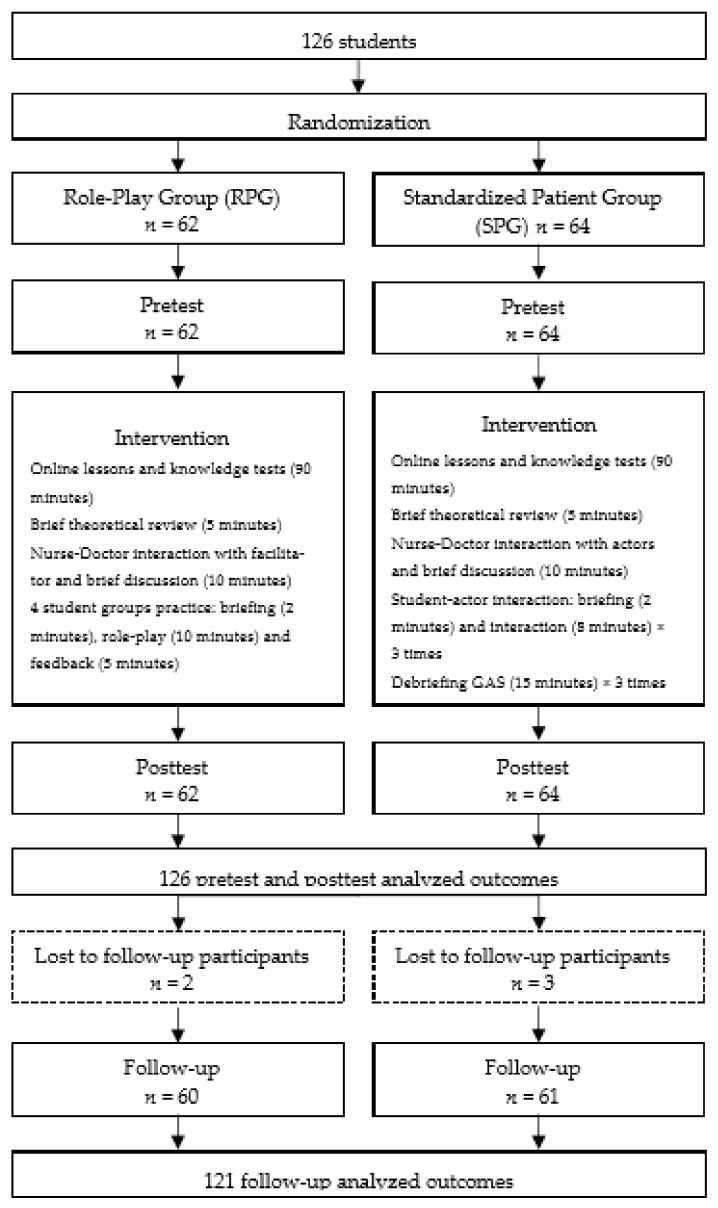
Protocol for randomisation and intervention.

A controlled clustered randomised trial was designed (Figure 1). Students were randomised into one of two training groups: the standardised patient simulation group (SPG) or the role-play group (RPG).

### 2.2. Sample/Participants

Participants’ eligibility criteria were: (1) to be enrolled in the nursing degree at the University of Almería (Spain) and (2) not to have received training in interprofessional communication. The sample size was calculated a priori using a conservative approach. Thus, to compare two proportions with a 95% confidence interval and 80% power to detect significant statistical differences (*p* < 0.05), it was estimated that a sample of 94 subjects was needed. To compensate for possible attrition, an extra 25% was added to this estimation, and so the sample size was set at 118 students. Eventually, a total of 126 students voluntarily participated in the study, of whom five were lost to follow-up because they could not complete the pre-test, post-test or follow-up measures. The demographic data collected were: age, sex, educational level and previous interprofessional communication training.

### 2.3. Data Collection

The study was carried out in the elderly care module during the third year of the nursing degree program at the University of Almería (Spain). Before enrolling in the study, the participants had already completed 6 weeks of clinical placement in hospital wards and nursing homes. In the faculty where the study was conducted, third-year students are divided into teaching groups composed of 16-18 students at the beginning of the academic year. The faculty’s administrative staff alphabetically allocates the students into eight teaching groups. For the current study, each teaching group was blindly assigned a numerical code (1–8) and randomly assigned to the SPG or RPG using a free online randomisation service.

The summarised intervention protocol can be seen in Figure 1. Both groups (SPG and RPG) received a 3 h intervention that included both 1.5 h of online training (video-recorded lessons and formative knowledge test) and a 1.5 h face-to-face workshop based on either standardised patient simulation or role-play (see Figure 1).

Both groups started the face-to-face workshop with a brief review of the main concepts pertaining to interprofessional communication in elderly care. Then, students observed and analysed a nurse–doctor interaction carried out by two actors (SPG) or two facilitators (RPG). Thereafter, the session was different for each group. SPG training was carried out with an actor who played a healthcare professional (elderly care consultant, house officer and nurse) in three different settings. A volunteer student for each scenario interacted with the actor while their peers observed them, and they all participated in the subsequent debriefing as recommended by the International Nursing Association for Clinical Simulation and Learning (INACSL) [41]. The debriefing followed the gather-analyse-summarise (GAS) method, which stands for gather information, analyse it in order to facilitate students’ reflections and summarise the lessons learned, providing a clear understanding to students [42]. In the RPG, students were divided into groups of four students and they worked on four case studies (one per group). One student played the nurse role, another student acted as an elderly care doctor or elderly care senior nurse and the other two students observed the interaction and gave feedback at the end (they started with the things that went well, then commented on things that could have been improved and finished with a positive comment to take forward). All groups rotated and students exchanged roles in each case study. The facilitator provided individual intermittent feedback to all participants in the RPG and moderated the debriefing in the SPG. In order to minimise bias, the same facilitator delivered all the training workshops. The facilitator had previous experience using role-play as a teaching method and had completed post-graduate courses on clinical simulation. All the scenarios used in the study are available on request from the corresponding author.

### 2.4. Instruments

The three domains of the competency were individually assessed for all participants before (pre-test), immediately after completing the workshops (post-test) and 6 weeks after the intervention (follow-up). To test the students’ skills, they had to interact with a previously trained actor in a simulated scenario while a researcher observed their performances. All assessments were videotaped and two researchers independently marked the participants’ interactions.

- The level of knowledge on interprofessional communication skills was assessed according to the interprofessional communication subscale of a multiple-choice questionnaire (IC-MCQ) [43]. The IC-MCQ comprised four questions about the situation-background-assessment-recommendation (SBAR) technique with five options and only one correct answer, including an ‘I don’t know’ answer.
- Psychomotor skills were assessed using the IC-Checklist [43]. The IC-Checklist comprised five items pertaining to the skills needed to exchange information with other healthcare professionals. Using a rubric, the items were rated on a scale of 0–5, from ‘not competent’ to ‘fully competent’.
- The level of self-efficacy was assessed with the ‘Patient clinical Information Exchange and interprofessional communication Self-Efficacy Scale’ (PIE-SES) from the ‘Clinical Communication Self-Efficacy Toolkit’ [44]. The PIE-SES comprised six items rated on a scale of 0–100, from ‘I’m sure I can’t do it’ to ‘I’m sure I can do it’.

### 2.5. Outcome Measures

Knowledge on interprofessional communication: following similar studies’ benchmarks and marking standards in the environment where the study was carried out, participants’ knowledge levels were deemed adequate when they achieved a score equal to or greater than 70% on the IC-MQ [45].

Skills in interprofessional communication: participants’ skills were considered adequate if an average score of 3 points or more was obtained in the IC-Checklist [46].

Self-efficacy in interprofessional communication: as recommended by other authors in studies with a similar methodology, participants were considered to have achieved a high level of self-efficacy in interprofessional communication when their score was equal to or greater than 70% in the PIE-SES [46,47].

Competence in interprofessional communication in elderly care: participants were considered to have achieved competence when they scored ≥ 70% in the IC-MCQ, ≥3 points in the IC-Checklist and ≥70% in the PIE-SES.

### 2.6. Ethical Considerations

The study was carried out at the University of Almería (Spain) between September of 2017 and February of 2018. The ethics and research committee of the Department of Nursing, Physiotherapy and Medicine granted ethical approval before starting the study and contacting the potential participants (EFM 10/15). Written detailed information was provided to all eligible subjects in order to inform them about the study’s aims and procedures, which included videorecording their performances for their subsequent evaluation. Furthermore, participants were asked to voluntarily sign an informed consent document before participating, confirming that they understood the information provided and their right to withdraw from the study at any time without any academic consequences. All data were processed in accordance with the European Data Protection Legislation, governed by Regulation (EU) 2016/679 of the European Parliament and the Council of 27 April 2016 [48].

### 2.7. Data Analysis

Data analysis was performed using IBM^®^ SPSS^®^ v.25 for Windows^®^. A descriptive analysis of sociodemographic variables was carried out. Key baseline demographic variables were compared between groups using independent *t*-tests for continuous data and chi-squared tests for categorial data. In order to study the effect of the interventions, the frequency and percentage of students who reached the benchmark on each domain, as well as for competence in interprofessional communication, were calculated at the pre-test, post-test and 6-week follow-up points. Between-subject differences were tested using the chi-Squared test. Within-subject differences were tested using the McNemar test. For both analyses, studied differences were considered to be statistically significant if the *p*-value < 0.05. Furthermore, a generalised estimating equation (GEE) analysis with logit link function was used to compare the differences in counts and proportions of students who achieved competence after participating in the SPG or the RPG. In this case, Bonferroni correction was applied and differences were considered statistically significant if *p*-values < 0.025.

## 3. Results

### 3.1. Sample Characteristics

Participants’ demographic characteristics and data about previous basic interpersonal communication training are presented in Table 1.

Participants’ average age was 22.53 years (SD = 6.36) and the sample comprised 77.0% (*n* = 97) female participants. In addition, 97.6% (*n* = 123) had completed upper secondary education before entering the nursing degree and 64.3% (*n* = 81) had received previous training in basic interpersonal communication. Homogeneity of sample characteristics was assumed as long as there were no statistically significant differences between groups (SPG and RPG) (Table 1).

### 3.2. Intervention Outcomes

Table 2 summarises the counts and proportion of participants who achieved the benchmark for knowledge, skills, self-efficacy and overall interprofessional communication in elderly care competence in both groups (SPG and RPG) at the pre-test, post-test and follow-up points. GEE analysis showed that between-group differences across time were not statistically significant for any of the studied variables (*p* > 0.05). In order to further explore the effects of each intervention on participants’ acquisition and retention of interprofessional communication in elderly care competence, we carried out pairwise comparisons for all the studied variables.

Table 3 shows the results of comparing the learning improvement from pre-test to post-test amongst both groups. The McNemar’s test results showed that participants’ knowledge, skills, self-efficacy and overall competence in interprofessional communication in elderly care significantly improved in both the SPG and the RPG (*p* < 0.05). The level of knowledge, skills, self-efficacy and overall competence retention was assessed by analysing the differences in the proportion of participants who achieved the benchmark between the post-test and the follow-up (see Table 4). The McNemar’s test results showed that there was not a significant decrease in knowledge, skills and overall competence for either the RPG or the SPG (*p* > 0.05). Lastly, Table 5 shows the differences in the proportion of participants who achieved the benchmark for all the studied variables at pre-test and follow-up for both groups. According to McNemar’s test, there were significant differences between pre-test and follow-up measures on the count of participants who achieved the benchmark for knowledge, skills, self-efficacy and overall competence in both groups (*p* < 0.05).

When comparing the success rates of both groups (SPG and RPG) using the chi-square test, no significant differences were observed at either pre-test, post-test or follow-up on any of the studied variables (see Table 3 and Table 4).

## 4. Discussion

This study aimed to describe and compare the effects of two teaching strategies (standardised patient simulation vs role-play) in the acquisition and retention of interprofessional communication in elderly care competence amongst nursing students. Both strategies resulted in a significantly higher proportion of students achieving a good level of knowledge, skills, self-efficacy and competence in interprofessional communication in elderly care. However, no significant between-group differences were found for any of the studied variables at any time.

Role-play and standardised patient simulation have been reported to be efficient strategies to teach non-technical skills to nursing students [34,35]. A combination of lectures and solving case studies that involve assuming different roles has shown an improvement for communication skills and confidence in healthcare team interactions [16,39,49]. This improvement has also been found in several studies that have used standardised patient simulation as a teaching method [20,40,50,51,52]. However, this study seems to be the first one that proposes a comparison of the effects of these two teaching methods on nursing students’ competence (knowledge, skills and self-efficacy) in interprofessional communication in elderly care.

Regarding knowledge in interprofessional communication in elderly care, our results showed there were not any statistically significant differences between using standardised patient simulation and role-play. This concurs with a previous study carried out by Kesten [39], and it could be explained by the idea that any intervention promotes a greater awareness about what students need to know in order to improve their communication with other team members [11,53,54]. Furthermore, group dynamics and the feedback received from peers (RPG) or through the debriefing (SPG) may have contributed to solidifying participants’ knowledge through individual reflection, group discussion and self-evaluation [4,30,55].

Regarding self-efficacy, our results showed a significant increase in the proportion of students who achieved the predefined benchmark between pre-test and post-test after both interventions. However, there were not any statistically significant differences in students’ level of self-efficacy when comparing the effects of both methods. These outcomes are supported by several studies that use standardised patient simulation [20,40,50,51,52,56] or role-play [16,49]. Having used both modelling at the beginning of the workshops and peer-learning methods could have contributed to these results [57]. The standardised patient simulation and role-play methods provided a safe environment where making mistakes did not imply serious consequences for the students, so this could have made them less fearful when interacting with other members of the multidisciplinary team looking after older adults [58,59,60]. Furthermore, students only had had one clinical placement before the intervention where they merely focused on shadowing their allocated nurse and perform basic tasks, and they had not had any previous experience at communicating clinical information about older people to other healthcare professionals themselves. As the present study took place right before starting their second clinical placement, this could have resulted in a strong motivation for learning and a boost for their self-efficacy [30,52,54,61]. In addition, some studies [49,50,62,63] have reported that experiential learning methods make students focus on the way they carry out their interactions and this influences their self-efficacy scores.

In terms of skills, our results also showed a significant improvement between pre-test and post-test, after both role-play and standardised patient simulation interventions. These results concur with other studies that implement one of these methods [16,40,52]. The evidence suggests that these learning methods encourage a better understanding of the roles which could facilitate the improvement of students’ performance, as long as they know what to do and how to do it [64]. In fact, the assumption of different roles makes students capable of developing adequate decision-making, communication, and teamwork skills [64]. Furthermore, both interventions were strictly structured and they included student–lecturer interaction and feedback from classmates, which are both known to be learning facilitators and which could have influenced the students’ skill scores [4,30,40].

Regarding overall competency, our results showed that more than 50% of the students in both SPG and RPG achieved a good level of competence. These outcomes could be explained by several factors. On the one hand, various activities based on observation, skills performance and feedback were used in both interventions. These activities promote a better integration of the competency and improve students’ skills [46]. In fact, peer observation and feedback may have helped students to notice and to minimise mistakes during their practice [47,65]. Along with this, these activities involve self-directed training, which may have increased the students’ motivation to learn and may have given them the opportunity to self-monitor their progress and to make changes to improve their skills [47,57]. On the other hand, both interventions were carried out in a laboratory whose characteristics were not equal to a clinical setting or elderly care unit. This fact could have made students uncomfortable, reducing the efficacy of the strategies [54,62,66]. Moreover, the workshops were short in duration, which made it difficult to train in various settings, reducing the opportunities for students to learn through their own experiences and peer observation [30,40,54,67]. In addition, for interprofessional communication in elderly care, it is mandatory to have clinical knowledge in order to exchange adequate information and to apply recommendations. Therefore, those students who did not have a minimum level of clinical knowledge, could have encountered more difficulties when trying to achieve a good level of competence [18]. Finally, students had not had any previous experience of exchanging clinical information about older people with other healthcare professionals, making the acquisition of these communication skills more complicated [52].

### Limitations

To the best of our knowledge, this is the first study that focuses on analysing and comparing the effects of standardised patient simulation and role play in the acquisition and retention of competence in interprofessional communication in elderly care among nursing students. However, there are some limitations that may restrict the generalisability and interpretation of our results. First, the study was conducted in a local university, there was no randomisation on the selection of the participants and they met very specific inclusion criteria which means that our results cannot be generalised to populations with different characteristics. Secondly, both interventions included a combination of activities, so it is not possible to identify the real effect of each activity on the participants’ results. Third, students did not have previous experience in standardised patient simulation. Although they were informed about the method and video-recorded sessions on the dynamics of this method were available to the students through the online teaching platform, not having participated in standardised patient simulation before could have led them to feel stressed and anxious, thereby, affecting their performance [30,67,68]. Forth, no formal pre-briefing was performed in order to establish a safe environment and this could have conditioned students’ performance and results [69]. Fifth, previous studies have not set a clear, evidence-based benchmark for what can be considered to be an appropriate level of self-efficacy, knowledge or skills in interprofessional communication. In this regard, we arbitrarily set up the benchmark at 70% for all variables, which is in line with the recommendation with the marking systems in our environment. Last, we could only include a 6-week follow-up. Consequently, we cannot ascertain how long the participants’ knowledge, self-efficacy, skills and overall competence would remain above the pre-defined benchmark.

## 5. Conclusions

Educational interventions using standardised patient simulation or role-play as a teaching method have been shown to improve knowledge, skills, self-efficacy and competence in interprofessional communication amongst nursing students in elderly care. Based on the study results, the use of standardised patient simulation does not lead to more students acquiring and retaining knowledge, skills, self-efficacy and competence in interprofessional communication when compared to using role-play as a teaching method. Future research should focus on conducting larger studies with longer interventions, and medium-term follow-ups should be carried out. Furthermore, future research should also study participants’ stress or anxiety levels as well as motivation and satisfaction, since they are described as factors that can influence students’ performance.

## Figures and Tables

**Table 1:** Demographic characteristics of the study sample.

Characteristics	SPG (*n* = 64)	RPG (*n* = 62)	Total Sample (*n* = 126)	*t*-Test	*p*-Value
	*M* ± SD	*M* ± SD	*M* ± SD		
Age (years)	22.77 ± 6.70	22.29 ± 6.03	22.53 ± 6.36	−0.44	0.66
	***n (%)***	***n (%)***	***n (%)***	**Χ^2^**	***p*-value**
Gender Female Male	51 (79.7) 13 (20.3)	46 (74.2) 16 (25.8)	97 (77.0) 29 (23.0)	0.54	0.46
Education level (completed) Upper secondary education Others	62 (96.9) 2 (3.1)	61 (98.4) 1 (1.6)	123 (97.6) 3 (2.4)	2.48	0.48
Basic interpersonal communication training	36 (56.2)	45 (72.6)	81 (64.3)	0.06	0.80

**Table 2:** Counts (proportions) of participants who achieved the benchmark for all variables measuring competence in interprofessional communication and GEE analysis.

	SPG			RPG			Time vs. Int.
	Pre-Test (*n* = 64)	Post-Test (*n* = 63)	Follow-Up (*n* = 63)	Pre-Test (*n* = 62)	Post-Test (*n* = 61)	Follow-Up (*n* = 61)	*p* ^1^
Knowledge							
≥70% of IC-MCQ answered correctly	4(6%)	46(72%)	40(62%)	7(11%)	41(66%)	46(74%)	0.54
Self-efficacy							
≥70% achieved in PIE-SES	29(45%)	47(76%)	48(77%)	30(48%)	52(85%)	50(82%)	0.56
Communication Skills							
≥3 points achieved in IC-Checklist	27(42%)	62(98%)	60(97%)	31(50%)	59(97%)	57(93%)	0.99
Interprofessional Communication Competence							
Overall competence achieved ^2^	1(2%)	36(56%)	35(55%)	2(3%)	38(63%)	42(68%)	0.48

^1^ GGE analysis: *p*-value in time vs intervention group interaction. Significance is reached at 0.025, according to the Bonferroni correction = 0.05/2. ^2^ Interprofessional communication competence = ≥70% of IC-MCQ answered correctly; ≥70% achieved in PIE-SES; and ≥3 points achieved in IC-Checklist.

**Table 3:** Counts (proportions) of dichotomous interprofessional communication competency parameters per group for pre-test and post-test.

	SPG			RPG			SPG vs. RPG Pre-Test	SPG vs. RPG Post-Test
	Pre-Test *n* = 64	Post-Test *n* = 63	*p*-Value ^1^	Pre-Test *n* = 62	Post-Test *n* = 61	*p*-Value ^1^	*p*-Value ^2^	*p*-Value ^2^
Knowledge								
≥70% of IC-MCQ * answered correctly	4(6%)	46(72%)	<0.001	7(11%)	41(66%)	<0.001	0.48	0.49
Self-efficacy								
≥70% in PIE-SES ** achieved	29(45%)	47(76%)	<0.001	30(48%)	52(85%)	<0.001	0.53	0.20
Communication skills								
≥ 3 points achieved in IC-Checklist	27(42%)	62(98%)	<0.001	31(50%)	59(97%)	<0.001	0.42	0.54
Interprofessional communication competence								
Overall competency achieved ^3^	1(2%)	36(56%)	<0.001	2(3%)	38(63%)	<0.001	0.29	0.45

* IC-MCQ is the multiple-choice questionnaire used to assess cognitive knowledge on interprofessional communication. ** PIE-SES is the scale used to measure participants’ self-efficacy in interprofessional communication.^1^ McNemar test. Significance is reached at 0.05. ^2^ Chi-squared test. Significance is reached at 0.05. ^3^ Interprofessional communication competence = ≥70% of IC-MCQ answered correctly; ≥70% achieved in PIE-SES; and ≥ 3 points achieved in IC-Checklist.

**Table 4:** Counts (proportions) of dichotomous interprofessional communication competency parameters per group for post-test and follow-up test.

	SPG			RPG			SPG vs. RPG Follow-Up Test
	Post-Test *n* = 63	Follow-Up Test *n* = 63	*p*-Value ^1^	Post-Test *n* = 62	Follow-Up Test *n* = 61	*p*-Value ^1^	*p*-Value ^2^
Knowledge							
≥70% of IC-MCQ * answered correctly	46(72%)	40(62%)	0.24	41(66%)	46(74%)	0.30	0.16
Self-efficacy							
≥70% in PIE-SES ** achieved	47(76%)	48(77%)	1	52(85%)	50(82%)	1	0.56
Communication skills							
≥ 3 points achieved in IC-Checklist	62(98%)	60(97%)	1	59(97%)	57(93%)	0.69	0.38
Interprofessional communication competence							
Overall competency achieved ^3^	36(56%)	35(55%)	1	38(63%)	42(68%)	0.61	0.13

* IC-MCQ is the multiple-choice questionnaire used to assess cognitive knowledge on interprofessional communication. ** PIE-SES is the scale used to measure participants’ self-efficacy in interprofessional communication. ^1^ McNemar test. Significance is reached at 0.05. ^2^ Chi-squared test. Significance is reached at 0.05. ^3^ Interprofessional communication competence = ≥70% of IC-MCQ answered correctly; ≥70% achieved in PIE-SES; and ≥3 points achieved in IC-Checklist.

**Table 5:** Counts (proportions) of dichotomous interprofessional communication competency parameters per group for pre-test and follow-up test.

	SPG			RPG		
	Pre-Test *n* = 63	Follow-Up Test *n* = 63	*p*-Value ^1^	Pre-Test *n* = 62	Follow-Up Test *n* = 61	*p*-Value ^1^
Knowledge						
≥70% of IC-MCQ * answered correctly	4(6%)	40(62%)	<0.001	7(11%)	46(74%)	<0.001
Self-efficacy						
≥70% in PIE-SES ** achieved	29(45%)	48(77%)	0.004	30(48%)	50(82%)	<0.001
Communication skills						
≥3 points achieved in IC-Checklist	27(42%)	60(97%)	<0.001	31(50%)	57(93%)	<0.001
Interprofessional communication competence						
Overall competency achieved ^2^	1(2%)	35(55%)	<0.001	2(3%)	42(68%)	<0.001

* IC-MCQ is the multiple-choice questionnaire used to assess cognitive knowledge on interprofessional communication. ** PIE-SES is the scale used to measure participants’ self-efficacy in interprofessional communication. ^1^ McNemar test. Significance is reached at 0.05. ^2^ Interprofessional communication competence = ≥70% of IC-MCQ answered correctly; ≥70% achieved in PIE-SES; and ≥3 points achieved in IC-Checklist.
